# Interstellar Ices: A Factory of the Origin-of-Life Molecules

**DOI:** 10.1021/acscentsci.3c01636

**Published:** 2024-01-09

**Authors:** Cristina Puzzarini, Silvia Alessandrini

**Affiliations:** Department of Chemistry “Giacomo Ciamician”, University of Bologna, Via F. Selmi 2, I-40126 Bologna, Italy

In the past few years, molecular discovery in space has made tremendous progress in terms of both the number of identified molecules and their complexity. Out of 305 molecular species detected in space since 1937, 84 (∼30%) have been identified in the last 3 years, while the observed molecules that contain 10 or more atoms have nearly reached the milestone of 50 compounds. (See the Cologne Database). Although the evidence for molecular complexity is undisputed, there is still much to be understood about the formation of molecules under the extreme conditions of the interstellar medium (ISM) and their possible connection to the origin of life on earth. Kaiser, Chang, and co-workers^1^ took an important step toward such a direction. In their work recently published in *ACS Central Science*, they demonstrated that, in the interstellar grains, already at low temperatures (below 100 K), carbamic acid (H_2_NCOOH, **1**) is formed from ammonia (NH_3_) and CO_2_ as reactants without the help of any energetic radiation. At even lower temperatures, ammonium carbamate ([H_2_NCOO^–^][NH_4_^+^], **2**) starts to form.^1^ This finding is particularly significant because carbamic acid is the simplest species containing both the carboxyl (−COOH) and amino (−NH_2_) groups, a characteristic they share with amino acids. Indeed, glycine (the simplest amino acid) differs from carbamic acid only for an additional CH_2_ group inserted between the carboxyl and amino moieties. To understand its biological relevance, it suffices to say that the conjugated phosphate, carbamoyl phosphate (H_2_NCOOPO_3_), is the first step in the fixation of ammonia toward the synthesis of pyrimidine-derived nucleobases and a couple of amino acids (arginine and glutamine). The conjugated ammonium salt **2** can be considered to be a prototype molecule for studying the reactivity of more complex carbamates, which play an important role in biochemistry (for example, functionalized carbamates are involved in the fixation of CO_2_). Carbamic acid and its ammonium salt can be seen as a reservoir for the amino (−NH_2_), ammonium (NH_4_^+^), carboxylic (−COOH), and carboxylate (−COO^–^) moieties. Their delivery to newly formed terrestrial environments via meteorites and comets could have been a plausible source of prebiotic molecules on the early earth. Thus, elucidating the synthesis of both **1** and **2** surely provides new insights into the abiotic origin of biorelevant molecules in the ISM and might shed some light on the origin of life on earth.

**Figure 1:**
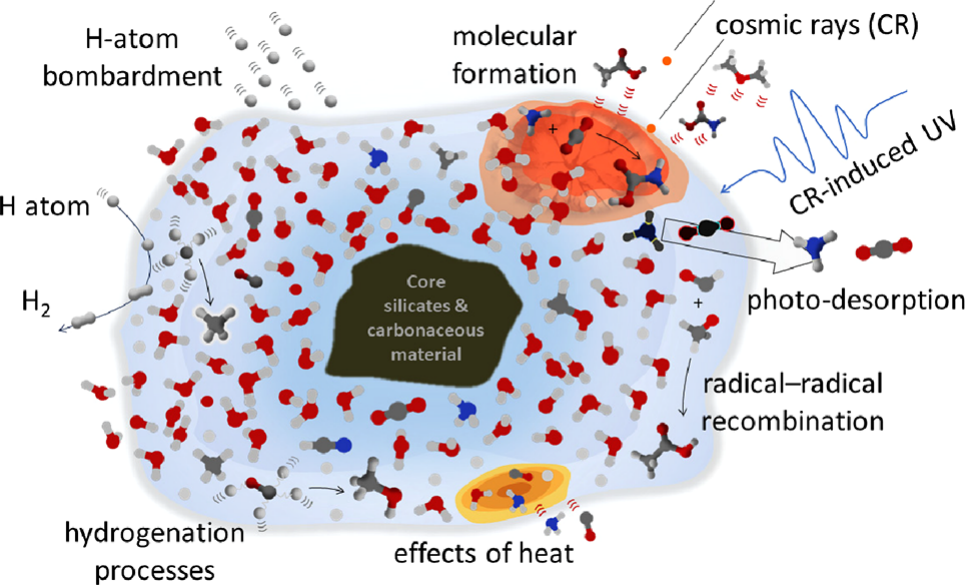
An interstellar dust grain: a chemical factory. Schematic representation of its composition and reactivity.

Let us take a step back in the narrative. The ISM, the matter between the star systems in a galaxy, is not homogeneous, and its conditions are extreme: the temperature ranges between 10 and 200 K, the density is very low (from 1 to 10^8^ particle·cm^–3^), and ionizing radiation is present. Interstellar matter consists of gas (∼99%) and dust particles (∼1%) and is concentrated in the so-called “clouds”. Dust grains (typically about 0.1 μm in diameter) are composed of silicates and carbonaceous compounds. In dense clouds (also denoted as molecular clouds, characterized, on average, by temperatures of about 10 K and densities of 10^2^–10^7^ particle·cm^–3^), this grain core is surrounded by water ice containing various molecules such as CO, CO_2_, CH_4_, NH_3_, and CH_3_OH. Figure 1 provides a pictorial representation of a dust particle, also pointing out its reactivity. Indeed, interstellar dust grains can be considered to be a chemical factory. The low temperatures that are typical of the molecular clouds put severe constraints on chemical reactivity: assuming that the adsorbed atomic and molecular species can diffuse on the ice surface, only barrierless reactions can occur, which should be furthermore characterized by mechanisms presenting only submerged barriers.^2^ However, suprathermal chemical reactions can take place within icy mantles thanks to irradiation (galactic cosmic rays and UV photons)^3−5^ and lead to the formation of the so-called complex organic molecules (COMs),^6^ which are considered to be key precursors and building blocks of biological molecules (such as amino acids, sugars, DNA bases, etc.).

In cold molecular clouds (∼10 K), interstellar dust grains are covered by water ice. This is the result of a long-lasting *in situ* process leading to the accretion of a thick (up to more than 100 layers) amorphous icy mantle. At the same time, atoms and molecules colliding with interstellar grains are adsorbed on them, where they may diffuse and react, thus enriching the chemical composition of the grain mantle. Typically, adsorption is due to van der Waals forces or electrostatic interaction (physisorption). The phenomena occurring on and inside the icy mantle are of paramount importance from an astrochemical point of view: they are, indeed, the key to understanding the chemical composition of the ISM. Most of the species frozen on or trapped inside the grain mantles are observable once released into the gas phase because of thermal- or photo-desorption or sputtering of the mantel (in shocked regions).^7^ Therefore, the laboratory simultaneous analysis of the condensed and gas phases, as carried out by Kaiser, Chang, and co-workers,^1^ allows for gaining important insights into the competing processes of decomposition, dimerization, and sublimation. Indeed, it permits us to answer a crucial question: will the COMs formed on the grain survive the desorption process? In ref (1), such an analysis pointed out the stability of **1** along with its dimer in the gas phase. Kaiser, Chang, and co-workers demonstrated that carbamic acid and its ammonium salt are potential candidates for astronomical observations. Both of them can be searched in the solid phase using, for example, the James Webb Space Telescope. For the former, future observations in the gas phase, possibly in star-forming regions where temperatures are ideal for its production and sublimation, should also be considered.

**Figure 2:**
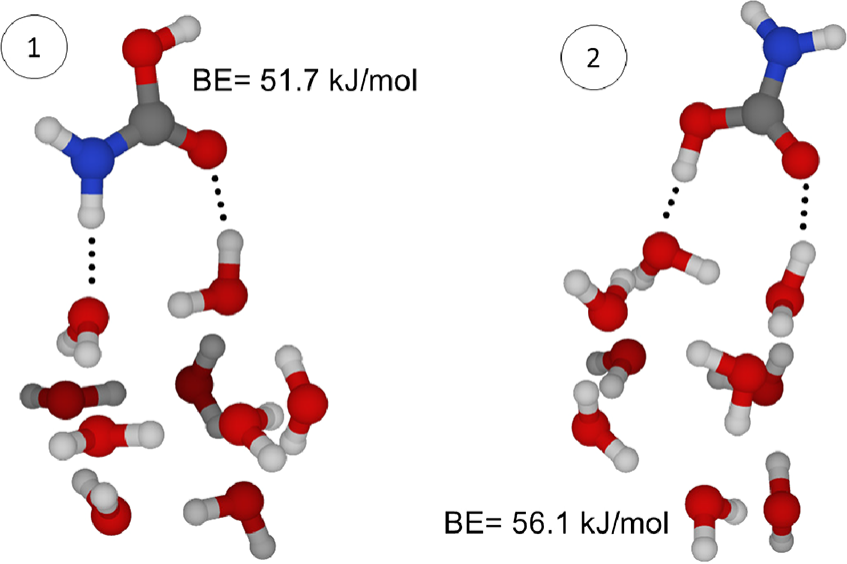
Carbamic acid adsorbed on a water icy dust mantel (amorphous ice model): BE depends on the interactions established (ZPE-corrected values at the B3LYP/jun-cc-pVDZ level of theory).

Desorption is governed by a key parameter, the binding energy (BE), which quantifies the strength of the interaction between the molecule and the icy mantel: the higher the BE, the higher the temperature required to thermally desorb. Consequently, BE values are important input parameters in astrochemical models and provide information not only on desorption but also on diffusion processes, which in turn impact the chemical reactivity. Both temperature-programmed desorption laboratory experiments and quantum-chemical calculations usually provide only a single value for the BE of the molecule under consideration. However, the grain mantel being amorphous leads to a large variety of possible adsorption sites, and the molecular species can show different orientations (and thus different interactions). A simplified representation is provided in Figure 2 for **1**: even considering a nonrepresentative model for an amorphous water ice mantel, it is evident that **1** can interact differently with the H_2_O molecules and BE changes noticeably. Consequently, not a single BE value but rather a distribution of BEs needs to be evaluated, thus requiring the development of *ad hoc* strategies (e.g., ref (8)).

As mentioned above, Kaiser, Chang, and co-workers^1^ suggested **1** as a good candidate for radioastronomical detection. Let us again take a step back in the narrative. Radiotelescopes collect the electromagnetic radiation (in the microwave region) that reaches earth, which is due to the gas-phase chemical species present in the portion of the ISM under observation.^7^ The outcomes are the so-called radioastronomical spectra, which are usually extremely complex because of overlapping features due to tens (or even hundreds) of molecules. Consequently, the identification of **1** in the ISM requires its accurate spectroscopic characterization in the laboratory in order to search for its fingerprints in the “radioastronomical-spectra forest”.^9,10^ More generally, what is the guidance toward new discoveries? Laboratory studies, such as the one presented by Kaiser, Chang, and co-workers, able to reproduce the interstellar conditions and couple the gas- and condensed-phase compositions, pave the way for understanding the chemical complexity in space. How far does the chemical complexity in space go? Is the chemistry of the ISM related to the origin of life? To tackle these challenging questions, the synergism of different laboratory investigations is required, with studies such as the one in ref (1) providing crucial guidance.
